# Income Disparities and Nonresponse Bias in Surveys of Patient Experience

**DOI:** 10.1007/s11606-020-05677-6

**Published:** 2020-01-31

**Authors:** Brian W. Roberts, Jady Yao, Christian J. Trzeciak, Louis S. Bezich, Anthony Mazzarelli, Stephen Trzeciak

**Affiliations:** Cooper University Health Care and Cooper Medical School of Rowan University, Camden, NJ USA

## INTRODUCTION

Recent research suggests an absence of meaningful healthcare disparities in patient experience for disadvantaged populations as assessed by Hospital Consumer Assessment of Healthcare Providers and Systems (H-CAHPS)—the patient survey platform used by the Centers for Medicare and Medicaid Services (CMS).^1, 2^ However, the data sources only contained data for returned surveys, and response rates were unavailable. Thus, the potential for nonresponse bias, where responders are not typical of the target patient population (e.g., disadvantaged persons), could not be assessed. Our objective was to assess patient experience survey response rates across socioeconomic status (SES) strata and to test if patient experience surveys are at risk for nonresponse bias.

## METHODS

We analyzed 1 month (May 2019) of data for the Clinician and Group (CG)-CAHPS survey of patient experience for outpatient clinic visits at Cooper University Health Care (CUHC), an academic health system with > 100 ambulatory care locations in New Jersey. The CG-CAHPS survey was sent to all patients who had an outpatient clinic visit and had a mailing address or email on file. We determined survey response rate for each municipality (according to the patients’ home address) in the CUHC catchment area. We excluded municipalities with total population < 5000 persons and/or < 300 sent surveys based on number needed for reliable estimates.^3, 4^ We abstracted data for median annual household income, race/ethnicity, and education levels for each municipality from Census Bureau Population Estimates.^3^ We used linear regression (weighted by number of surveys sent in each municipality) to test if median annual household income—calibrated for $10,000 increments—predicts response rate. We also tested if median annual household income was associated with a gap (relative difference) between the education level of survey respondents and Census Bureau Population Estimates. Finally, we tested if municipality annual household income was associated with overall patient experience (using ordered logistic regression), and access to care (i.e., able get an appointment for care you needed right away (yes/no)) (using logistic regression) among respondents.

## RESULTS

**Figure 1:**
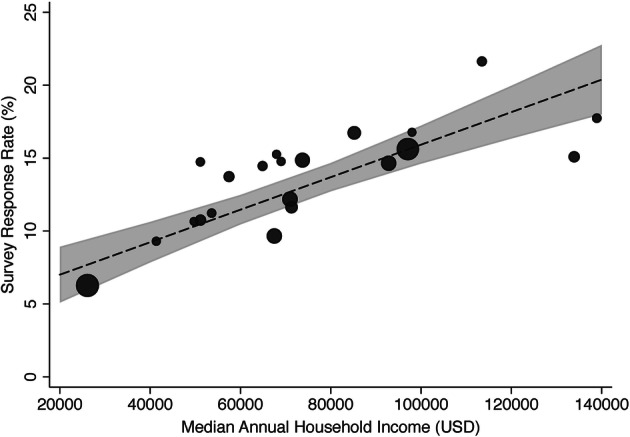
**Survey response rate and median annual household income for 21 different municipalities. The size of the data points represents the number (*N*) of surveys sent. Dotted line represents the fitted regression line and the shaded area represents 95% confidence intervals. USD, United States dollars.**

Twenty-one municipalities were included. Consistent with similar out-patient experience survey response rates (e.g., ~ 12%),^5^ our overall response rate was 12.8% (2081/16,272). Figure 1 displays survey response rate by household income across a wide range of income disparity. Lower median annual household income was associated with lower survey response rate (*β* = 1.1 (95% CI 0.8 to 1.4, *p* < 0.001)). The municipality with the highest median annual household income ($138,920) was 91.0% white and had a survey response rate of 17.7% (66/372). The proportion of survey respondents with a bachelor’s degree was similar to the Census Bureau estimate, 67.0% versus 74.7%, relative difference − 10%. The municipality with the lowest median annual household income ($26,105) was only 5.9% white and had a survey response rate of 6.3% (168/2680). The proportion of survey respondents with a bachelor’s degree was more than twice the Census Bureau estimate, 19.0% versus 8.3%, relative difference 130%. Lower median annual household income was associated with higher relative difference in proportion of survey respondents with a bachelor’s degree compared with Census Bureau data (*β* = − 0.1 (95% CI − 0.2 to − 0.03, *p* < 0.001)). We did not find household income to be associated with overall patient experience (*β* = − 0.004 (95% CI − 0.04 to 0.04, *p* = 0.844)) or access to care (odds ratio 1.04 (95% CI 0.94 to 1.14, *p* = 0.463)).

## DISCUSSION

We found that in low SES populations, survey response rates are exceptionally low, and patients that do respond are more likely to have graduated from college than what would be expected from Census Bureau estimates. We also did not find an association between SES and overall patient experience or access to care. These results suggest nonresponse bias, and thus conventional survey methodology may not accurately assess patient experience in disadvantaged populations.

We acknowledge limitations. We studied a single healthcare system. We also did not have income data at the individual patient level and used aggregate data at the municipality level instead. Although the overall survey response rate was low, it is consistent with patient experience survey response rates from similar studies.^5^

In conclusion, nonresponse bias may affect patient experience surveys in low SES populations. Further research is needed to identify optimal methodologies for assessing patient experience among disadvantaged persons.
